# Population-Based Biochemistry, Immunologic and Hematological Reference Values for Adolescents and Young Adults in a Rural Population in Western Kenya

**DOI:** 10.1371/journal.pone.0021040

**Published:** 2011-06-21

**Authors:** Clement Zeh, Pauli N. Amornkul, Seth Inzaule, Pascale Ondoa, Boaz Oyaro, Dufton M. Mwaengo, Hilde Vandenhoudt, Anthony Gichangi, John Williamson, Timothy Thomas, Kevin M. DeCock, Clyde Hart, John Nkengasong, Kayla Laserson

**Affiliations:** 1 U.S. Centers for Disease Control and Prevention (CDC-Kenya), Kisumu, Kenya; 2 Centre for Global Health Research, Kenya Medical Research Institute/U.S. CDC Research and Public Health, Kisumu, Kenya; 3 Department of Internal Medicine, Center for Infection and Immunity (CINIMA), Center for Poverty-Related Communicable Diseases (CPCD), Academic Medical Center, Amsterdam Institute for Global Health and Development (AIGHD), University of Amsterdam, Amsterdam, The Netherlands; 4 Institute of Tropical Medicine (ITM), Antwerp, Belgium; 5 Global AIDS Program, National Center for HIV, Viral Hepatitis, STD, and TB Prevention, U.S. Centers for Disease Control and Prevention, Atlanta, Georgia, United States of America; University of Toronto, Canada

## Abstract

**Background:**

There is need for locally-derived age-specific clinical laboratory reference ranges of healthy Africans in sub-Saharan Africa. Reference values from North American and European populations are being used for African subjects despite previous studies showing significant differences. Our aim was to establish clinical laboratory reference values for African adolescents and young adults that can be used in clinical trials and for patient management.

**Methods and Findings:**

A panel of 298, HIV-seronegative individuals aged 13–34 years was randomly selected from participants in two population-based cross-sectional surveys assessing HIV prevalence and other sexually transmitted infections in western Kenya. The adolescent (<18 years)-to-adults (≥18 years) ratio and the male-to-female ratio was 1∶1. Median and 95% reference ranges were calculated for immunohematological and biochemistry values. Compared with U.S-derived reference ranges, we detected lower hemoglobin (HB), hematocrit (HCT), red blood cells (RBC), mean corpuscular volume (MCV), neutrophil, glucose, and blood urea nitrogen values but elevated eosinophil and total bilirubin values. Significant gender variation was observed in hematological parameters in addition to T-bilirubin and creatinine indices in all age groups, AST in the younger and neutrophil, platelet and CD4 indices among the older age group. Age variation was also observed, mainly in hematological parameters among males. Applying U.S. NIH Division of AIDS (DAIDS) toxicity grading to our results, 40% of otherwise healthy study participants were classified as having an abnormal laboratory parameter (grade 1–4) which would exclude them from participating in clinical trials.

**Conclusion:**

Hematological and biochemistry reference values from African population differ from those derived from a North American population, showing the need to develop region-specific reference values. Our data also show variations in hematological indices between adolescent and adult males which should be considered when developing reference ranges. This study provides the first locally-derived clinical laboratory reference ranges for adolescents and young adults in western Kenya.

## Introduction

An increasing number of clinical trials taking place in sub-Saharan Africa are seeking to identify safe and effective prevention and treatment strategies to combat the heavy burden of infectious diseases in this region [1], [2]. Africa is disproportionately affected by numerous viral, parasitic and bacterial diseases, including: 66% of the global HIV/AIDS infections [3], [4], 31% of the tuberculosis infections, and 86% of the malaria cases [5]. Clinical trials in sub-Saharan Africa need accurate clinical laboratory reference ranges for appropriate screening of volunteers in clinical trials, monitoring disease progression, and evaluating possible clinical trial-associated toxicity and adverse events. Traditionally, normal ranges for clinical laboratory values have been obtained mainly from European and North American populations [2]. However, differences are known to occur between normal Africans values with those of North Americans and Europeans [6]. For example, African populations are reported to have lower hemoglobin (HB), red blood cells (RBCs), hematocrit (HCT), mean corpuscular volumes (MCV), platelets and neutrophils, and higher monocyte and eosinophil levels than their Western counterparts [6]–[9]. Moreover, there are variations in indices between different African ethnic groups [9]–[12]. Factors such as genetics, dietary patterns, gender, age, ethnic origin and environmental pathogens are known to influence hematological and immunologic indices [13]–[16]. Thus, the use of normal laboratory values derived from external populations could produce selection bias leading to exclusions of otherwise healthy volunteers in clinical trials, misclassification of adverse events, and a framework for allowing incorrect patient management in routine clinical care.

Besides the relevant utility of laboratory reference values for clinical trials, such values are also important in routine health assessment, particularly for screening of anemia, blood disorders and diseases of the immune system. Of particular importance is the use of these indices as surrogate markers for disease progression and response to anti-retroviral therapy in HIV-infected individuals [17]. Decisions to initiate, monitor, or change antiretroviral therapy (ART) regimens are determined using CD4+ T-lymphocyte cell (CD4) counts, while drug toxicity is monitored using liver function tests (LFT) and renal function tests (RFT), and complete blood counts (CBC) [18], [19]. Due to differences in these parameters between Western and African populations, it is necessary to develop a range of local values for these indices. In addition, differences in hematological and lymphocyte indices between age groups also suggests the need to develop age-specific reference ranges [8], [14], [15], [20]. However, information about reference values based on age groups is limited for the Africa populace [6], [21]–[23].

The aim of our study was to generate normal ranges of laboratory values for blood chemistry, hematologic and immunologic indices of healthy, HIV-negative females and males aged 13 to 34 years. We also determined gender and age variations in these indicators. In addition, we compared our normal range values with those previously reported from the African continent and with North American-derived reference ranges from the Massachusetts General Hospital [24] and the U.S. NIH Division of AIDS (DAIDS) which are currently used to measure the severity of adverse events in most clinical research studies [25].

## Methods

### Study population and subject recruitment

Between October 2003 and May 2005, two baseline cross-sectional surveys were conducted by the US Centers for Disease Control and Prevention (CDC) in collaboration with the Kenya Medical Research Institute (KEMRI), the Institute of Tropical Medicine in Antwerp (ITM), and the London School of Hygiene and Tropical Medicine (LSHTM) in two rural communities on the shores of Lake Victoria about 50 kilometers from Kisumu city in western Kenya. The aim of the surveys was to estimate the prevalence of HIV and sexually transmitted infections (STIs), and associated risk factors, in preparation for a future biomedical HIV-prevention intervention trial in rural western Kenya [26].

The majority of the inhabitants in this area are of Luo ethnic group (98%). This region is ∼3700 feet above sea level, has perennial malaria transmission and both endemic schistosomiasis and helminthic infections [27], [28]. KEMRI/CDC has a longstanding presence in this region through malaria research and a comprehensive health and demographic surveillance system (HDSS) covering approximately 220,000 residents [29].

The first survey in Asembo, Rarieda District, enrolled 1822 individuals aged 13 to 34 years via stratified random sampling by gender and age-group; the second survey in the Gem Siaya District used cluster sampling (villages) to enroll 912 individuals aged 15 to 34 years. In both surveys, all participants received a review of medical history, a physical examination, testing for HIV and pregnancy (for females), and treatment for medical illnesses and STI that were diagnosed [26]. Participants were included in the two surveys if permanent residents of the area, between 13 and 34 years of age and able to provide informed consent or assent in case of minors.

From the two surveys, approximately 11% of blood samples from clinically healthy participants in each of the two districts were selected to generate reference ranges in this study. Participants were excluded if HIV-seropositive, pregnant, exhibiting febrile symptoms and on medication. Selection of participants from the two surveys involved stratified systematic sampling with strata comprising of males and females and further sub-stratification by age (i.e. adolescents; <18 vs. adults; ≥18) to include an approximate equal number of participants in each sub-strata. After stratification samples from participants who met the study eligibility criteria were systematically included in the analysis in each stratum until the required number was achieved for each sub-stratum.

### Ethical approval

Ethical approval for the study was obtained from KEMRI/CDC, ethics review committee/institutional review board. Written informed consent was obtained from each participant prior to study initiation. Patients also consented for the use of their stored samples for other future studies which included the reference ranges analysis. Minors (<18 years of age) were classified as “mature” or “non-mature” using legal definitions. Mature minors were married, a parent, or a head of household and could consent to study participation as they would for HIV counseling and testing in Kenya [30]. Non-mature minors went through a two-step written consent process involving consent from the parent or guardian followed by a private discussion with the minor that included a thorough explanation of the study after which written consent was obtained from those willing to participate.

### Blood collection and HIV serology

Whole blood was collected in vacutainer tubes containing EDTA (Becton Dickinson, Franklin Lakes, NJ) and transported to the KEMRI/CDC HIV-research laboratory for processing and analysis within six hours of specimen collection. The HIV status was determined from whole blood using HIV rapid test kits as follows: Determine (Abbot Laboratories, Tokyo, Japan), and Unigold (Trinity Biotech Plc, Bray, Ireland), with Capillus (Trinity Biotech Plc, Bray, Ireland) as a tie breaker.

### Pregnancy testing

A urine pregnancy test was administered to all females of child-bearing age, who were not visibly pregnant, using Randox, Inc. latex monoclonal agglutination test (Antrim, Northern Ireland, UK), or First Sign HCG One Step (UNIMED International, Inc., South San Francisco, CA, USA).

### Hematological analysis

Absolute white blood cell counts and percentages for leukocytes (WBC) with differentials (neutrophils, lymphocytes, monocytes, eosinophils, and basophils), erythrocytes (RBC) with parameters (hemoglobin, hematocrit, MCV, and MCH), and platelet counts were determined from whole blood using a Coulter ACT 5Diff CP analyzer (Beckman Coulter, France). This was performed within 24 hours of sample collection as recommended by the manufacturer.

### Flow cytometry analysis of lymphocyte subsets

Lymphocyte subsets were analyzed from whole blood on a FACS calibur flow cytometer (Becton Dickinson, San Jose, CA) with the following combinations of monoclonal antibodies (MAb): anti-CD3-fluorescein isothiocyanate (FITC), anti-CD45-Peridinin chlorophyll protein (PerCP), anti-CD4-Allophycocyanin (APC), and anti-CD8-Phycoerythrin (PE) as per the manufacturer's recommendations. Staining was done within 12 hours of blood collection. Analysis was done within 24 hours after staining and the results were checked using the MultiSET software (Becton Dickinson, San Jose, CA).

### Biochemistry analysis

Clinical chemistries from serum samples were analyzed for aspartate aminotransferase (AST), alanine aminotransferase (ALT), total bilirubin (T-bil), glucose (Glu), creatinine (Cr), and blood urea nitrogen (BUN) using the Cobas Integra 400 plus biochemistry analyzer (Roche, Germany) per the manufacturer's instructions.

### Quality Control

Quality control protocols included running known standards each day before testing samples. In addition, the laboratory is enrolled in external quality assurance testing programs with the College of American Pathologists (lymphocyte immunophenotyping, hematology, and clinical chemistry) and the United Kingdom National External Quality Assurance Service (lymphocyte immunophenotyping).

### Statistical analysis

Data were collected on optical character recognition (OCR) enabled forms and entered with scanners. The data were then grouped according to age (<18 years of age as adolescents and ≥18 as adults) and gender, and analyzed using SAS v9.1 (Cary, NC, USA). The normal distribution for each group was tested using the Kolmogorov-Smirnov test that provided the median and the 95^th^ percentile ranges. To eliminate bias due to small sample size, a bootstrap analysis method was used as a robust method to assess the 95% reference ranges from the Kolmogorov-Sminorv test. For each parameter, 10,000 bootstrap samples were selected and analyzed using SAS v9.1. [26], [31] and the lower 95% reference limit was defined as the 2.5 percentile while the upper limit was defined at the 97.5 percentile.

A Wilcoxon rank-sum test was used to test for differences between and within the two age groups. A two-sided P value of <0.05 was considered significant. Box and whisker plots were also plotted for parameters in which age variation existed to assess distribution of these variations with age.

We then compared our data against reference intervals from the Massachusetts General Hospital (MGH-USA) [24] and the U.S. NIH Division of AIDS (DAIDS) toxicity tables [25] to determine the number of study participants who had values outside the MGH ranges or who had any adverse event as graded by the DAIDS criteria. The Pearson chi-square test was used to compare the proportion of values outside the MGH ranges by gender and age categories.

## Results

A total of 2707 participants were enrolled in the two baseline cross-sectional surveys. Of these 2307 were HIV-seronegative. From these participants a total of two hundred and ninety eight specimens from participants who met the eligibility criteria were included in this study. One hundred and fifty four (51.7%) of these were males while 144 (48.3%) were females. Almost half (46%) of the study population were less than 18 years old, representing 77 (50%) of all males and 62 (47%) of all females.

### Hematological Parameters

The mean, median and 95% reference values stratified by age and gender, for different hematological parameters are presented in Table 1.

**Table 1:** Hematological reference values (median and 95^th^-percentile) stratified by age and gender from a 13–34 years old cohort in rural western Kenya (2003–2005).

	Age 13–17 years		Age 18–34 years	
Parameter	Male (n = 76)	Female (n = 57)	Male (n = 77)	Female (n = 83)
**RBC (10^6^ Cells/µl)**	4.9 (4.1–5.8)	4.7 (3.3–5.4)	5.3 (4.3–6.5)	4.5 (3.4–5.7)
**Hb (g/dL)**	13.1(10.6–15.6)	12.2 (8.1–14.2)	14.2 (11.4–16.9)	12.1 (8.0–14.2)
**HCT (%)**	38.8 (29.3–48.1)	35.6 (24.8–43.1)	41.7 (32.6–51.5)	35.8 (23.2–44.3)
**MCV (fL)**	79 (62–92)	78 (57–91)	80 (55–98)	79 (60–94)
**PLT (10^3^ cells/µl)**	224 (103–386)	233 (134–439)	201 (102–307)	220 (88–439)
**WBC(10^3^ cells/µl)**	5.6 (3.3–8.3)	5.2 (3.9–10.2)	5.3 (2.5–7.4)	5.6 (3.3–9.7)
**Ne (10^3^ cells/µl)**	1.9 (0.8–5.0)	2.0 (1.1–3.1)	2.0 (0.8–3.9)	2.3 (1.3–3.8)
**Ly (10^3^ cells/µl)**	2.2 (1.0–4.2)	2.2 (1.1–3.1)	2.2 (1.0–3.5)	2.2 (1.3–3.8)
**Mo (10^3^ cells/µl)**	0.5 (0.2–0.7)	0.4 (0.2–0.7)	0.5 (0.2–0.9)	0.5 (0.3–0.8)
**Eo (10^3^ cells/µl)**	0.4 (0.1–1.8)	0.4 (0.1–2.2)	0.5 (0.1–1.7)	0.4 (0.1–1.3)
**Ba (10^3^ cells/µl)**	0.04 (0.02–0.30)	0.04 (0–0.10)	0.04 (0.01–0.19)	0.04 (0–0.20)

#### Differences between age and gender groups

We observed statistically significant differences in RBC, HB and HCT by gender, with males having higher values than females in both age groups (Table 2).

**Table 2:** Test of difference in hematologic, clinical chemistry and immunologic parameters between gender and age-groups from the 13–34 years old rural western Kenya cohort (2003–2005).

	Age 13–17 years			Age 18–34 years			
Parameter	n	median	p-value (gender)	n	median	p-value (gender)	P-value(age)
**Hemoglobin (g/dL)**							
**Female**	57	12.2 (8.1–14.2)	***<.0001***	83	12.1(8.0–14.2)	***<.0001***	*0.3243*
**Male**	76	13.1 (10.6–15.6)		77	14.2 (11.4–16.9)		***<.0001***
**Hematocrit (%)**							
**Female**	57	35.6 (24.8–43.1)	***<.0001***	83	35.8 (23.2–44.3)	***<.0001***	*0.8015*
**Male**	76	38.8 (29.3–48.1)		77	41.7 (32.6–51.5)		***<.0001***
**WBC (×1000)**							
**Female**	57	5.2 (3.9–10.2)	*0.6359*	83	5.6 (3.3–9.7)	***0.0189***	*0.2038*
**Male**	76	5.6 (3.3–8.3)		77	5.3 (2.5–7.4)		*0.6382*
**RBC (×10^12^/L)**							
**Female**	57	4.7 (3.3–5.4)	***0.0001***	83	4.5 (3.4–5.7)	***<.0001***	*0.2638*
**Male**	76	4.9 (4.1–5.8)		77	5.3 (4.3–6.5)		***<.0001***
**Lymphocytes (×10^9^/L)**							
**Female**	57	2.2 (1.1–3.1)	*0.9820*	83	2.2 (1.3–3.8)	*0.6901*	*0.9388*
**Male**	76	2.2 (1.0–4.2)		77	2.2 (1.0–3.5)		*0.585*
**Ab Neutrophiles (×10^9^/L)**							
**Female**	57	2.0 (1.0–6.2)	*0.4991*	83	2.3 (1.3–5.4)	***0.0004***	*0.0576*
**Male**	76	1.9 (0.8–5.0)		77	2.0 (0.8–3.9)		*0.6575*
**PLT(×10^9^/L)**							
**Female**	57	233 (134–439)	*0.2958*	83	220 (88–439)	***0.0222***	*0.4034*
**Male**	76	224 (103–386)		77	201 (102–307)		***0.0094***
**AST/SGOT (µ/L)**							
**Female**	62	22.6 (12.0–43.1)	***0.0102***	82	22.2 (13.5–48.5)	*0.0822*	*0.5905*
**Male**	77	26.9 (17.0–59.2)		77	26.7 (12.5–69.3)		*0.9147*
**ALT/SGPT (µ/L)**							
**Female**	62	17.4 (4.2–65.3)	*0.6289*	82	18.9 (10.7–61.3)	*0.2247*	*0.1305*
**Male**	77	20.5 (4.9–42.4)		77	22.4 (12.0–80.6)		*0.0901*
**Total Bilirubin (µmol/L)**							
**Female**	62	9.7 (3.7–38.5)	***0.0331***	82	11.5 (5.8–36.1)	***0.0368***	*0.7132*
**Male**	77	13.9 (5.7–62.6)		77	13.8 (5.3–50.7)		*0.6662*
**Creatinine (µmol/L)**							
**Female**	62	64.5 (48.0–87.6)	***0.0229***	82	70.7 (52.4–96.8)	***<.0001***	***0.0013***
**Male**	77	66.3 (49.6–103.7)		77	83.1(54.2–137.8)		***<.0001***
**CD4: Absolute**							
**Female**	58	934 (465–1553)	*0.4074*	83	866 (440–1602)	***0.0141***	*0.509*
**Male**	76	874 (367–1571)		77	811 (462–1306)		***0.0209***
**CD8: Absolute**							
**Female**	58	506 (195–1068)	*0.4506*	83	472 (262–1167)	*0.8706*	*0.9213*
**Male**	76	468 (195–988)		77	468 (201–1104)		*0.4194*
**CD4/CD8 ratio**							
**Female**	58	1.8 (0.9–3.2)	*0.9215*	83	1.8 (0.8–3.0)	*0.0728*	*0.4879*
**Male**	76	1.8 (0.8–2.8)		77	1.6 (0.8–2.8)		*0.0543*

**Figure 1:**
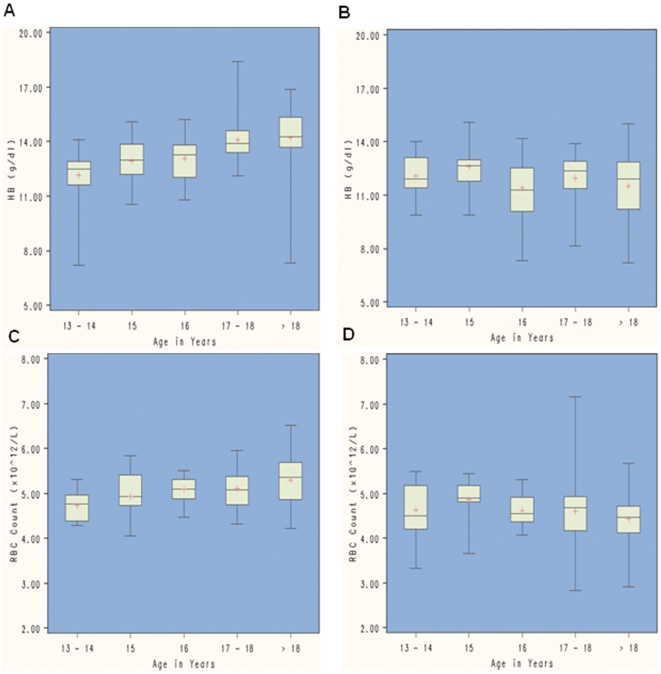
Box and whisker plots showing variation in hematological values with age and gender. Hemoglobin (A and B) and Red Blood Cell count (C and D) variation with age in both males (left panel) and females (right panel) from rural western Kenya.

We also observed differences in the hematological indices among males by age, with the young adults having higher levels of HB, HCT, RBC, and PLT as compared to adolescents P<0.001) (Table 2). This variation was observed as a progressive increase with age from adolescents to young adults as seen in the box and whisker plots (Figure 1a & c). In comparison, there was little variation in the same parameters among adolescents and adult females (Figure 1b & d).

Platelet counts were significantly higher among young adult females compared to the males in the same age group (P = 0.0222) and also differed between adolescent and young adult males (P = 0.0094) (Table 2). The median and 95th percentile values for absolute and WBC subsets (neutrophils, lymphocytes, monocytes, eosinophils and basophils) stratified by both age and genders are shown in Table 1. No gender or age differences were observed in absolute lymphocytes, basophil, eosinophil and monocytes counts. There were significant differences in neutrophil counts between male and female young adults, with the females having higher counts than males (Table 2).

#### Comparison with MGH and other African cohort-derived values

Our hematological parameters showed significant differences from the MGH US-population-derived values (Table 3). A higher proportion of our study participants (22.5–35%) had WBC, neutrophil, HB, HCT and platelet counts that were outside the lower range of MGH US population-derived values. Eosinophil values, however, were significantly higher, and the upper range in our study participants was 3.75 fold as high. These differences were distributed between age and gender with no statistically significant difference except for hemoglobin, where the younger population had slightly more out-of-range values (OOR) (53%) than the older group (34%).

**Table 3:** Out of range and frequency of adverse events in the rural western Kenyan cohort obtained from comparison with values from DAIDS and North American derived MGH values.

				out of range Comparison			Division of AIDS toxicity grading (DAIDS) [26]							
							Grade 1		Grade 2		Grade 3		Grade 4	
Parameter	n	Units	Study Reference intervals	USA [25]	n	%	n	%	n	%	n	%	n	%
**Hemoglobin Males**	140	g/dl	10.8–16.1	13.5–17.5	65	46	2	1.3	0	0	2	1.3	0	0
**Hemoglobin Females**	153	g/dl	8.0–14.2	12–16	61	40	11	7.9	8	5.7	14	10	0	0
**Hct (females)**	140	%	23.2–44.2	36–46	74	53								
**Hct (males)**	153	%	29.4–49.3	41–53	88	58								
**RBC (males)**	140	×10∧6 cells/µl	4.2–6.3	4.5–5.9	29	19								
**RBC (females)**	153	×10∧6 cells/µl	3.3–5.6	4.0–5.2	32	23								
**MCV**	293	fL	60–93	80–100	157	54								
**Platelets**	293	×10∧3 cells/µl	103–390	150–350	53	18	6	2	6	2	0	0	0	0
**WBC**	293	×10∧3 cells/µl	3.3–9.3	4.5–11.0	66	23	2	0.7	0	0	0	0	0	0
**Lymphocyte count**	293	×10∧3 cells/µl	1.1–3.5	1.0–4.8	6	2	0	0	0	0	0	0	0	0
**Neutrophil count**	293	×10∧3 cells/µl	0.9–5.2	1.8–7.7	110	38	25	8.5	9	3.1	1	0.3	0	0
**Eosinophil**	293	×10∧3 cells/µl	0.1–1.7	0–0.5	130	44	60	20.5	12	4.1	0	0	0	0
**Basophil count**	293	×10∧3 cells/µl	0.02–0.18	0–0.2	5	2								
**Monocyte count**	293	×10∧3 cells/µl	0.2–0.8	0–0.8	0	0								
**ALT (SGPT)**	293	IU/µl	7.2–61.3	0–35	30	10	12	4.1	1	0.3	0	0	0	0
**AST (SGOT)**	293	IU/µl	13.8–50.4	0–35	40	13	9	3.1	3	1	0	0	0	0
**Total Bilirubin**	293	µl mol/L	5.1–40.7	5.1–17.0	90	30	37	12.7	27	9.2	4	1.4	1	0.3
**Creatinine**	293	µl mol/L	50–113	0–133	4	1	4	1.4	0	0	0	0	0	0
**Glucose**	293	mmol/L	2.1–6.6	4.2–6.4	210	72								
**BUN**	293	mmol/L	1.2–5.1	3.6–7.1	246	84								
* **CD4**	293	Cells/µl	444–1488	404–1612	6	2	3	1	1	0.3	0	0	0	0
* **CD8**	293	Cells/µl	211–1078	220–1129	13	4								

*Reference ranges provided by Becton-Dickinson with the MultiTEST IMK Kit Reagent package (12/2000;23-3602-02).

-DAIDS- Division of AIDS tables for grading the severity of adult and pediatric adverse events [26].

- MGH-Massachusetts General Hospital weekly case records [25].

Comparing these values with those obtained from neighboring regions, the lower and upper limits for the hematological ranges obtained from this population were slightly lower than those derived from the Uganda, Tanzania and Ethiopia cohorts but were higher than those reported from the Kericho-Kenyan cohort, a region neighboring this study population (Table 4). However, age-specific data were not available for the comparison reference intervals.

**Table 4:** Hematological and Biochemistry Laboratory reference ranges derived from rural Western Kenya compared to other sources in Africa.

Parameter	Western-Kenya	Kericho-Kenya (2008) [6]	Uganda (2008) [47]	Tanzania (2008) [23]	Ethiopia (1999) [22]	Combined study from Kenya, Uganda, Zambia and Rwanda (2009) [37]
**Hemoglobin** (g/dL)						
Male	10.8–16.1	8.3–11.3	11.6–17.1	13.7–17.7	13.9–18.3	12.2–17.0
Female	8.0–14.2	5.9–10.0	9.8–16.2	11.1–15.7	12.2–16.1	9.5–15.8
**Hematocrit (%)**						
Male	29.4–49.3	40–50	33.8–49.5	40.2–53.7	41.6–55.1	35.0–50.8
Female	23.20–44.2	30–50	28.3–46.8	36.2–46.8	35.3–48.8	29.4–45.4
**RBC's** (10∧6 cells/µl)						
Male	4.2–6.3	4.4–6.3	3.8–6.1	4.4–6.3	4.3–5.9	4.0–6.4
Female	3.3–5.6	3.7–5.6	3.3–5.3	3.8–5.6	3.7–5.2	3.8–5.6
**Platelets** (10∧6 cells/µl)	103–390	120–411	109–384	150–395	N/A	126–438
**MCV** (fl)	60–93	68.8–97.2	71–97	77.6–98.1	N/A	68–98
**WBC** (10∧6 cells/µl)	3.3–9.3	2.8–8.2	2.8–8.2	2.7–8.3	3.0–10.2	3.1–9.1
**Neutrophils** (10∧3 cells/µl)	0.9–5.2	0.9–4.7	0.9–3.9	1.1–4.7	N/A	1.0–5.3
**Lymphocytes** (10∧3 cells/µl)	1.1–3.5	1.1–3.5	1.2–3.7	1.1–3.0	N/A	1.2–3.7
**Monocytes** (10∧3 cells/µl)	0.2–0.8	0.1–0.6	0.2–0.7	N/A	N/A	0.2–0.78
**Eosinophils** (10∧3 cells/µl)	0.1–1.7	0.03–1.1	0.04–1.60	N/A	N/A	0.04–1.53
**Basophils** (10∧3 cells/µl)	0.02–0.18	0.01–0.08	0.01–0.08	N/A	N/A	0.01–0.15
**CD4** (Cells/µl)	444–1488	421–1550	N/A	406–1392	366–1235	457–1628
**CD8** (Cells/µl)	211–1078	210–1081	N/A	188–990	311–1618	230–1178
**CD4∶CD8**	0.8–3.0	0.9–3.3	N/A	0.8–3.2	0.4–2.4	N/A
**Chemistries**						
**ALT** (IU/µl)	7.2–61.3	8.6–47.0	6.6–42.8	0–48.8	__	8–61
**AST** (IU/µl)	13.8–50.4	13.1–45.3	12.3–34.8	0–48	__	14–60
**T-Bil** (µl mol/L)	5.1–40.7	4.4–41.9	__	5.2–41	__	2.9–37
**Creatinine** (µl mol/L)	50–1488	__	__	__	__	
Male	52–125	62–106	__	__	__	47–109
female	49–97	51–91	__	__	__	47–109
**Glucose** (mmol/L)	2.1–6.6	3.1–5.7	N/A	2.9–5.2	__	N/A

### Immunological parameters

The mean, median and 95% reference values stratified by age and gender, for absolute and percentage CD4 and CD8 as well as the ratios are presented in Table 5.

**Table 5:** Lymphocyte sub-sets reference ranges (median and 95^th^-percentile) from a cohort (13–34 years) in rural Western Kenya (2003–2005).

	Age 13–17 years		Age 18–34 years	
Parameter	Male (n = 76)	Female (n = 58)	Male (n = 77)	Female (n = 83)
**CD4 (10^3^ cells/mm^3^)**	874 (367–1571)	934 (465–1553)	811 (462–1306)	866 (440–1602)
**CD8(10^3^ cells/mm^3^)**	468 (196–988)	505 (195–1068)	486 (201–1104)	472 (262–1167)
**CD4 %**	42 (32–56)	44 (30–56)	41 (29–54)	44 (32–55)
**CD8 %**	23.1(12.4–36.4)	23.5 (17.0–34.8)	24.6 (14.9–44.0)	24.3 (17.5–35.0)
**CD4∶CD8 ratio**	1.8 (1.0–3.1)	1.8 (0.9–3.2)	1.6 (0.8–2.8)	1.8 (0.8–2.8)

#### Differences between gender and age groups

Analyses of lymphocyte subsets indicated minimal differences between both gender and age groups, with the exception of CD4 indices. In both age groups, females had higher percentage and absolute CD4 cell count than males, the difference being significant only in the older age group. In assessing age variability, younger age was associated with higher CD4 cell counts and a higher CD4∶CD8 ratio, the difference being significantly higher in males (especially for CD4 counts) (Table 2).

#### Comparison with the US and other African cohort derived values

In comparing the upper and lower reference values from this population to the US-derived machine reference values, the CD4 cell counts were similar with only 2% being out of range (Table 3). Moreover, the upper limits of our overall CD4 cell counts closely compared with those reported from Mbeya in Tanzania, Kampala in Uganda and Kericho in Kenya (Table 4).

### Clinical Chemistry Parameters

The mean, median and 95% reference values stratified by age and gender, for clinical chemistry parameters are presented in Table 6.

**Table 6:** Clinical Chemistries Medians and 95% Reference Intervals stratified by age and gender from a 13–34 years cohort study in rural western Kenya (2003–2005).

	Age 13–17 years		Age 18–34 years	
Parameter	Male (n = 77)	Female (n = 62)	Male (n = 77)	Female (n = 82)
**ALT (U/L)**	20.5 (4.9–42.4)	17.4 (4.2–65.3)	22.4 (12.0–80.6)	18.9 (10.7–61.3)
**AST (U/L)**	26.9 (17.0–59.2)	22.6 (12.0–43.1)	26.7 (12.5–69.3)	22.2 (13.5–48.5)
**T-Bil (umol/L)**	13.9 (5.7–62.6)	9.7 (3.7–38.5)	13.8 (5.3–50.7)	11.5 (5.8–36.1)
**Creatinine (umol/L)**	66.3 (49.6–103.7)	64.5 (48.0–87.6)	83.1 (54.2–137.8)	70.7 (52.4–96.8)
**Glu (mmol/L)**	3.8 (2.2–6.6)	3.8 (2.0–7.0)	3.7 (2.1–9.0)	3.8 (2.1–6.0)
**BUN (mmol/L)**	2.5 (1.7–4.1)	2.3 (1.2–4.8)	3.0 (1.8–5.3)	2.8 (1.4–4.5)

#### Differences between gender and age groups

Analyses of liver and kidney function tests indicated gender and age variations between young adults and adolescents. Males had higher values for ALT, AST, T-bil and creatinine than females in both age groups, with those differences being significantly greater for T-bil and creatinine indices in both age-groups and AST among the adolescents. There were no gender differences in blood urea nitrogen and glucose levels for all age groups and no significant differences in T-bil, AST, ALT and glucose between the two age groups for both males and females. However young adult men and women did have higher values for creatinine and blood urea nitrogen compared to adolescent males and females, respectively.

#### Comparison with MGH and other African cohort derived values

Our chemistry reference intervals differed with the US MGH ranges. Of significance were the high proportions of participants with OOR values for BUN, glucose and T-bil which were 84%, 72% and 30% respectively (Table 3). There were no significant differences in the distribution of OOR values by age. Our values were comparable with those reported in other African cohorts, although our upper values for ALT and creatinine were higher than the other published studies (Table 4).

### Classification of Adverse Events using US-derived DAIDS Grading Criteria

Applying our data to the classification of adverse events using the DAIDS grading criteria for 11 of the indices in our study, over 40% of our otherwise healthy study participants would have erroneously been considered to have at least one laboratory-based grade 1–4 toxicity adverse event (AE) (Table 3). Values for eosinophil and neutrophil counts, HB, and LFT resulted in a majority of abnormal classifications. The high eosinophil counts observed would have led to 60 (20.5%) participants being classified as grade 1 and 12 (4.1%) as grade 2. Low HB levels among our participants would have resulted in 37 reported AEs; 13 (4.4%) as grade 1, 8 (2.7%) as grade 2 and 16 (5.5%) as grade 3. Low neutrophil counts would have resulted in 35 AE cases: 25 as grade 1 (8.5%), 9 (3.1%) as grade 2 and 1 as grade 3 (0.3%). The chemistry analytes also differed with the US-based values, especially for T-bil, where 68 participants would have been classified as having toxicity levels of grade 1 or above; 37 (12.7%) as grade 1, 27 (9.2%) as grade 2 and 4 as grade 3 (1.4%). DAIDS grading of our AST and ALT levels would also have classified a total of 25 grade 1–4 toxicity events with 12 (4.1%) and 9 (3.1%) as grade 1 and 1 (0.3%) and 3 as grade 2 (1.0%) for ALT and AST, respectively.

## Discussion

Clinical laboratory values provide important data to help assess the health of an individual. For this reason they are routinely used in clinical trials at enrollment and also during the course of the trial for monitoring the participants' health. Moreover, several analytes are used either as surrogate markers for indicating the possible presence of a disease or as direct evidence for that disease [32]–[34]. In the absence of locally derived reference values for African populations, clinicians and researchers have had to use reference values of European or North American populations. Previous studies have shown that such values vary with age, ethnic origin, socio-demographic characteristics, and environmental context [6], [8], [9], [21], [35]–[37]. The development of region and age-specific reference values is thus essential for efficient patient management and proper conduct of clinical research. This is especially critical for clinical trials being conducted in Africa to reduce the burden of such diseases as malaria, TB, and HIV in this region. Apart from their use in clinical trials, some laboratory markers are HIV disease surrogate markers and are thus important in care and treatment of HIV-infected patients in sub-Saharan Africa that has the greatest burden of the pandemic [4]–[6]. Our study was carried out in Nyanza Province, western Kenya, the region with the highest HIV prevalence in Kenya (15%) [38]. It is also the region in Kenya where many clinical research studies are being carried out [39]–[41]. Most of the values observed in this population differed with standard US-based reference values (MGH/DAIDS).

Regarding hematological indices, most of our values were lower than those derived from North American population; this finding is consistent with previous studies in other African regions [8], [21], [22]. However, our combined values for HB and MCV for both young adults and adolescents were higher than those reported from another study in Kericho, Kenya [6], but were slightly lower than those derived from Ethiopia, Kampala in Uganda and Mbeya in Tanzania [8], [21], [22]. The findings of significant gender differences in RBC parameters (RBC, HB, HCT, and MCV) are consistent with previously established evidence that men have higher values than females for these parameters. This difference is partly attributed to the influence of the androgen hormone on erythropoiesis [42], [43] and to menstrual blood loss in women [6], [8], [20], [21], [44]. As previously reported in other studies targeting adolescents [15], [20], older males from this population had significant higher values for RBC, HB and HCT than young males. The difference could be attributed to higher levels of androgen hormones among the older as compared to the younger males. This potential explanation is further supported by the absence of age-related hematological difference among females in our study, which is in agreement with findings obtained from previous studies targeting adolescent cohorts [15].

The lower platelet counts from this population as compared to Western values are synonymous with findings from other African studies [7], [8], [11], [22], [45]. The etiology of low platelet counts in African populations is unknown. However dietary, environmental and genetic factors have been proposed [7], [11], [45]. In agreement with other African studies [6], [8], [28], [35], [46] was the high eosinophil and low WBC and neutrophil values compared to those in North America. The eosinophilia may be attributed to increased parasitemia, since our study area is endemic for schistosomiasis, helminthic infections and perennial malaria [27], [28]. The low neutrophil count observed in our population could possibly be attributed to African genetics, environment or diet [36], [46].

The differences in gender and age in both WBC and CD4 cell counts are in agreement with previous reports [8], [14]. Females generally had higher counts, while young adolescents had equally higher values for both WBC and CD4 cell count as compared to adults. Overall our ranges for the lymphocyte subsets were higher and comparable to the USA reference ranges. The mean CD4 cell count for our population was 857.9, which is consistent with mean CD4 cell counts reported in other HIV-negative populations in Africa.

Liver and renal function tests are also important indicators of patient response to ARTs in the management of HIV/AIDS patients. Clinical chemistry laboratory reference values for LFT and renal function tests are limited in Africa, despite the continuous use of ARTs in this region. Most of our clinical chemistry reference ranges were comparable with the US MGH ranges except for T-bil and blood urea nitrogen. As seen from other studies conducted in Kenya [6], Uganda [8] and Tanzania [21] the upper range for T-bil seen in this study was twice as high as that of the US while the lower range for BUN was twice as low. The etiology of high T-bil in this population is thought to arise from a number of factors like RBC hemolysis caused by malaria or sickle cell disease, malnutrition or physical exertion. However the presence of similar trends among other African populations is suggestive of a common environmental or genetic factor [6], [37].

Analysis of the comparison between the values obtained from this population with those from the MGH ranges used in most clinical research studies revealed high variations for most values. If the US MGH derived ranges were used on this population during screening for any clinical research, over 58% of the volunteers would be screened out of the trial despite having laboratory results consistent with the general population. This erroneous screening would have important implications on study cost, work load and time, as more volunteers would be required for the screening process to meet the required target, even though the screening out process would actually have excluded healthy potential volunteers [47]. Moreover, the fact that the investigational product is designed for use in the same population in which the laboratory values differ with the values being used at the screening process might further complicate post-market analysis or adoption of the product for the general population.

Equally important is the comparison to the DAIDS toxicity tables; some of the ranges obtained extend between the normal and grade 1–2 toxicity grading. The lower range for HB (8.0 in females), neutrophil counts (0.9), as well as the upper range for eosinophil counts (1.68) and T-bil (40.7) would have been considered as grade 2 adverse events. In addition the observed lower ranges in males for HB (10.8) and the combined values for ALT (61.3) and AST (50.4) would have been classified as grade 1 adverse event. The use of the DAIDS toxicity grading for such populations may lead to inappropriate reporting of adverse events during clinical trials.

In agreement with other published data, we observed age-related variation between the adolescent males as compared to the adults for HB, HCT and RBC levels [8], [15], [20]. The fact that adolescents had lower hematological values is an issue that should be noted whenever clinical trials target this population. However, the observed differences may not be of medical significance, and thus there is need for further research as participation of adolescents in clinical trials increases. We observed no significant age differences in other parameters measured among males or in any parameters measured in females except for creatinine and BUN. This implies that adult values can be used in clinical trials involving adolescents for such parameters for which no differences were reported.

Several limitations could be cited in our study. Clinical Laboratory and Standards Institute (CLSI) guidelines for laboratory indicators recommend the consideration of genetic, environmental and social habits (such as smoking, dietary components, exercise and lifestyle) for which data were not obtained during enrollment of the participants in this study [11], [35], [37], [48]. The sample size was also small in the two age sets and did not meet the recommended CLSI sample size of 120 per every partitioned group [48]. However, a robust bootstrap analysis was used so as to eliminate bias as recommended by the Canadian laboratory initiative on paediatric reference intervals (CALIPER) [49]. In addition, although a thorough medical examination was done, not all sub-clinical conditions such as parasitic infections, hepatitis B infection or nutrition factors known to interfere with the obtained parameters were assessed. However, in the context of resource-limited settings, we assert that our study methods were sufficient to determine reference ranges for use in this population.

The findings from our study confirmed previously published data documenting differences in clinical laboratory reference ranges between African and Western populations. We also assert that there exist age variations in hematological values among males that reinforce the need to establish age-specific reference ranges for use in clinical trials involving such a population. This study presents the first description of biochemistry and hematological reference ranges in western Kenya, and is also the first study in the country that compares two distinct age sets between adolescents and adults. The development of these reference ranges may provide guidelines to be used by local health practioners in patient management within this region and for the design, conduct and evaluation of clinical trials for biomedical interventions. Whilst our study limitations may have influenced the different study parameters, our findings were comparable to those of other studies within Africa [6], [22], [23], [37], [47] and can be used as reference ranges for adolescents and young adults within Kenya.

As clinical trials and anti-retroviral treatment increase in Africa, locally derived clinical laboratory reference ranges are essential to ensure appropriate treatment monitoring, general health assessment and efficient execution of clinical trials. Similarly important is the need for development of toxicity grades for use among African populace in clinical care based on the differences observed between laboratory values from African population and the West. So far clinical studies as well as routine clinical patient management in most African countries are using either the European generated machine values, or the NIH division of AIDS toxicity grading in assessing critical values. The development of the various laboratory-derived African toxicity grades, in addition to the already developed reference values would thus be ideal for use in reporting adverse events in clinical trials as well as in routine health care for determining critical values. Using Western-derived reference ranges, a majority of our study participants would have been misclassified due to the high eosinophil counts and T-Bil as a consequence of regular exposure to endemic pathogens and inherent genetic factors leading to unnecessary treatment. Thus, physicians need to take into consideration the population limitations while attending to patients within this region. This study provides the first age-specific, locally derived clinical laboratory reference ranges in western Kenya for use in health care and clinical research.
